# Alterations in the histological features of the intestinal mucosa in malnourished adults of Bangladesh

**DOI:** 10.1038/s41598-021-82079-6

**Published:** 2021-01-27

**Authors:** Md. Shabab Hossain, S. M. Khodeza Nahar Begum, M. Masudur Rahman, Ramendra Nath Mazumder, Mashud Parvez, Md. Amran Gazi, Md. Mehedi Hasan, Shah Mohammad Fahim, Subhasish Das, Mustafa Mahfuz, Shafiqul Alam Sarker, Tahmeed Ahmed

**Affiliations:** 1 Nutrition and Clinical Services Division, International Centre for Diarrhoeal Disease Research, Bangladesh (icddr,b), 68, Shaheed Tajuddin Ahmed Sarani, Mohakhali, Dhaka, 1212 Bangladesh; 2 Department of Pathology, Bangladesh Specialized Hospital, Dhaka, 1207 Bangladesh; 3 Department of Gastroenterology, Sheikh Russel National Gastroliver Institute and Hospital, Dhaka, 1212 Bangladesh

**Keywords:** Gastroenterology, Medical research

## Abstract

There is paucity of knowledge on the histological features of the intestinal mucosa in malnourished adults of Bangladesh. The purpose of the study was to explore the histological features of the intestinal mucosa in malnourished adults of Bangladesh and to compare the findings with their well-nourished counterparts. 64 adults (37 malnourished with body mass index, BMI < 18.5 kg/m^2^ and 27 controls with BMI > 18.5 kg/m^2^) from the Bangladesh Environmental Enteric Dysfunction (BEED) study, who underwent upper-gastrointestinal endoscopy, were selected for this study. With a view to address the association of environmental enteric dysfunction (EED) with malnutrition, upper-gastrointestinal endoscopy was performed and mucosal biopsies from the distal duodenum were studied for histopathology. Villous height, crypt depth, and presence of inflammatory infiltrates in lamina propria were investigated. Bivariate analysis was performed to quantify the relation between malnutrition and the histological features. About 95% adults, irrespective of nutritional status, were diagnosed to have chronic non-specific duodenitis on histopathology. Malnourished adults suffered significantly more from chronic active duodenitis compared to their well-nourished counterparts (p = 0.003). Malnourished adults also had significantly higher frequency of subtotal villous atrophy, crypt hyperplasia and marked cellular infiltration in the lamina propria than the healthy controls (p < 0.05).

## Introduction

The gut is the central to health and development and a healthy ‘functional’ intestine enhances physical and intellectual development. In contrast, sub-optimal functioning of the gut leads to poor health^1^. The prevalence of chronic intestinal inflammation is very high in the tropics and it has been recognized that gut structure and function are almost universally abnormal, especially among children, living in these regions^2^. Alterations in small bowel function in children of developing countries in their early phases of life result from altered mucosal architecture and inflammation of the intestinal mucosa^3^. These changes are said to be the result of factors related to environmental contamination and persist throughout life. Studies show that, the condition has been reversed in non-native foreign individuals returning to their original homelands even after prolonged inhabitation in resource-poor settings, suggesting the role of exposure to contaminated environment^3–5^. Therefore, the altered mucosal architecture and inflammation of the intestinal mucosa is thought to be the consequence of chronic exposure to enteric pathogens through fecal contamination, and is being referred to as environmental enteric dysfunction (EED)^3^.

EED, previously known as environmental enteropathy (EE), predisposes to poor growth in children and malabsorption in people of all ages^6^ and is considered to be one of the major causative factors of malnutrition in this region^7^. The mechanism of EED has been described in many studies^8,9^. As EED is clinically asymptomatic, other than its manifestation as sub-acute weight loss and impaired growth, the traditional gold standard for diagnosis is considered to be intestinal biopsy and studying of the intestinal histology^10^. However, collection of small intestinal biopsy samples is ethically and technically infeasible, especially in asymptomatic individuals^8^. As a result, only a few studies conducted in some other countries have investigated this and have found shorter and thickened villi, increased crypt depth and inflammatory cellular infiltration in intestinal biopsy samples to be the hallmarks of EED^10^.

Though EED is acquired during infancy, it also persists in adulthood, and the fact is that it was actually first recognized in the adult population^11^. In general terms, the presentation pattern shifts from stunting to malabsorption induced malnutrition characterized by low body mass index (BMI). There is insufficient information on intestinal histological characteristics in malnourished adults of Bangladesh. Because of this knowledge gap, we sought to evaluate these parameters in malnourished adults and compare the findings with their well-nourished counterparts.

## Methods

### Study site and data collection

This sub-study, set up as a case–control design, is a part of the Bangladesh Environmental Enteric Dysfunction (BEED) study (ClinicalTrials.gov ID: NCT02812615; Link: https://clinicaltrials.gov/ct2/results?cond=NCT02812615&term=&cntry=&state=&city=&dist=; Date of first registration: 24/06/2016), which is a community-based nutrition intervention study conducted in Dhaka, Bangladesh. The objective of the BEED study was to validate non-invasive biomarkers of environmental enteric dysfunction (EED) with small intestinal biopsy in order to have a better understanding of the disease pathogenesis. In the adult cohort of the study, malnourished adults (BMI < 18.5 kg/m^2^) aged 18–45 years were enrolled. The enrolled subjects underwent thorough clinical assessment and laboratory investigations to exclude any organic disease or any other causes of secondary malnutrition including tuberculosis, diabetes mellitus etc. The participants received on-site nutritional intervention for 60 days consisting of one egg, 150 ml of whole milk and micronutrient sprinkles, as well as, nutritional counseling daily, for six days a week. The details, sample size calculation and overall design of the BEED study have already been published elsewhere^12^. Participants diagnosed with severe anemia (Hb% < 8 g/dl), tuberculosis and other chronic diseases including diabetes mellitus or any congenital disorder or deformity, pregnant or lactating women, drug abusers, known psychiatric disorders, cancer or other chronic or acute diseases and known allergy to any components of nutrition intervention were excluded. Enrolled participants failing to respond to nutritional therapy and also negative for secondary malnutrition, i.e. tuberculosis, parasitic infection as well as having no other clinical complaints were considered as probable cases of EED and upper-gastrointestinal (UGI) endoscopy was performed. As collection of small intestinal biopsy samples from absolutely healthy, well-nourished, asymptomatic individuals without an evident clinical condition is ethically infeasible, the control group considered in the present study consisted of age and sex matched well-nourished adults (BMI > 18.5 kg/m^2^) suffering from functional dyspepsia according to the Rome III criteria who underwent upper GI endoscopy for evaluation of dyspepsia. These control adults had no evidence of any organic diseases on endoscopy and were otherwise healthy, and were enrolled from the Gastroenterology Outpatient Department of Dhaka Medical College and Hospital (DMCH). The control participants who were diagnosed with peptic ulcer disease, malignancy etc. during the endoscopy procedure were excluded. For this particular study, a total of 64 adults (37 malnourished, 27 controls) were enrolled from July 2016 to May 2018. The study conforms to the principles outlined in the Declaration of Helsinki. Ethical approval was obtained from the Institutional review committee of icddr,b and all activities were performed in accordance with the relevant guidelines. The protocol number is PR-16007. A written informed consent for the intervention as well as UGI endoscopy and biopsy was obtained from the participants after a detailed explanation of the aims and procedures of the study.

### Duodenal biopsy

UGI endoscopy procedures of malnourished and control groups were performed by endoscopists (RNM and MMR) at icddr,b Dhaka Hospital and Dhaka Medical College Hospital, respectively, using Olympus CV-170 scopes under sedation. Biopsy specimens were taken from the distal part of the duodenum (distal to the ampulla of Vater) using EndoJaw FB-230 K 2.8 mm disposable biopsy forceps (Olympus Medical Systems Corporation, Tokyo, Japan). For fixation, biopsy samples were immediately placed in vials containing 10% buffered formalin solution and paraffin sections were prepared and stained by hematoxylin and eosin (H&E) stain. All the biopsies were reviewed by expert pathologists (KNB and MP), blinded to the case histories and endoscopic findings. The villous height, crypt depth, presence and intensity of inflammatory infiltrates in lamina propria and intraepithelial lymphocytes (IELs) were determined from the biopsied specimens^13^.

### Duodenitis and intestinal histological characteristics

**Figure 1:**
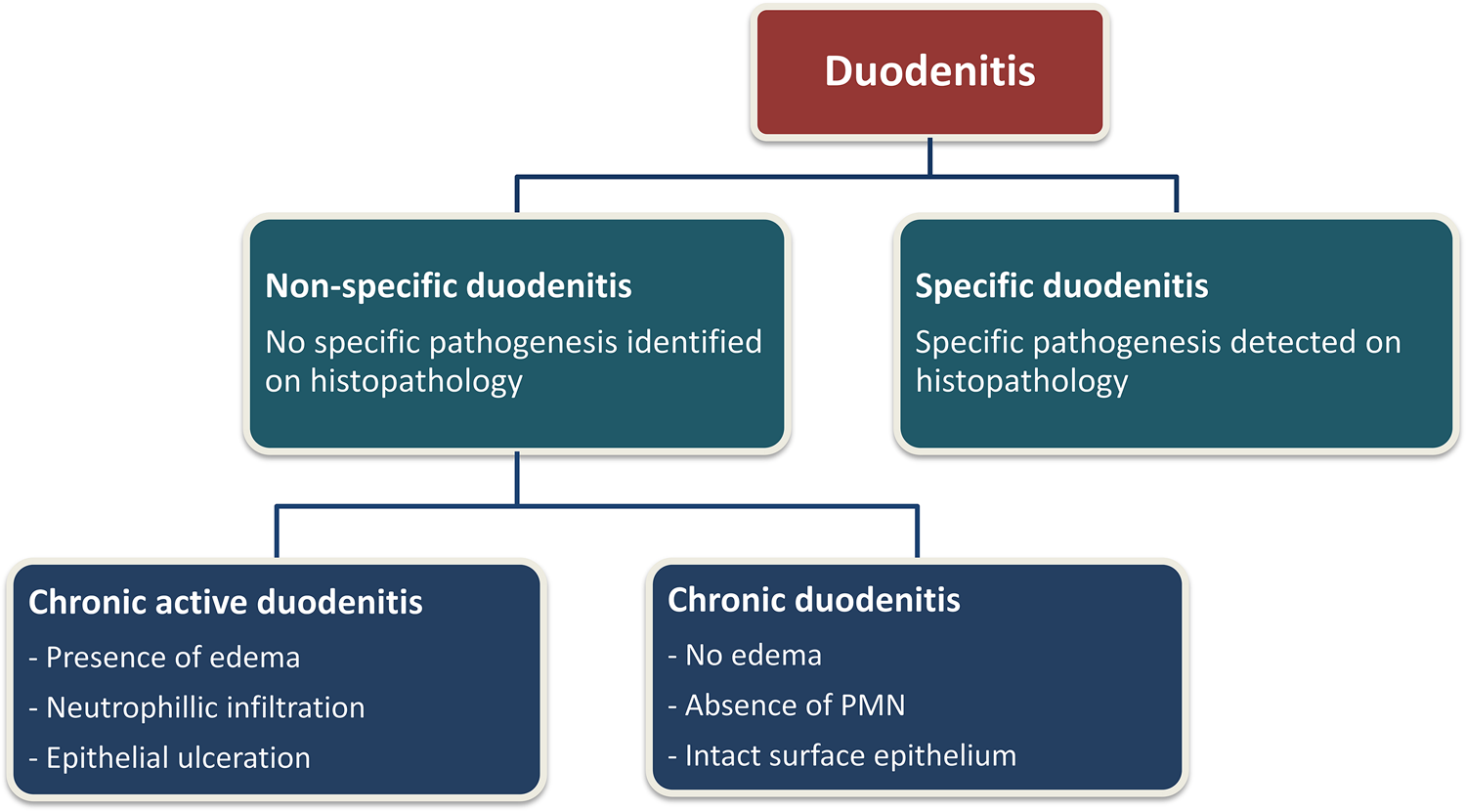
Operational classification of duodenitis based on pathogenesis and cellular activity. *Specific duodenitis* Presence of a disease process such as Crohn’s disease, sarcoidosis, etc.; *PMN* Polymorphonuclear leukocytes.

An operational classification of duodenitis was developed based on pathogenesis and cellular activity (Fig. 1). Non-specific duodenitis was defined as inflammation and morphological alteration of the duodenal mucosa not associated with any other pathological process^14^. Whereas, specific or secondary duodenitis referred to presence of a disease process such as Crohn’s disease, sarcoidosis, etc^15^. Whether the duodenitis was active (chronic active duodenitis) or not (chronic duodenitis) was based on the presence of neutrophilic infiltration or polymorphonuclear invasion, which is characterized by epithelial degeneration, regeneration and intercellular edema^16^.

**Figure 2:**
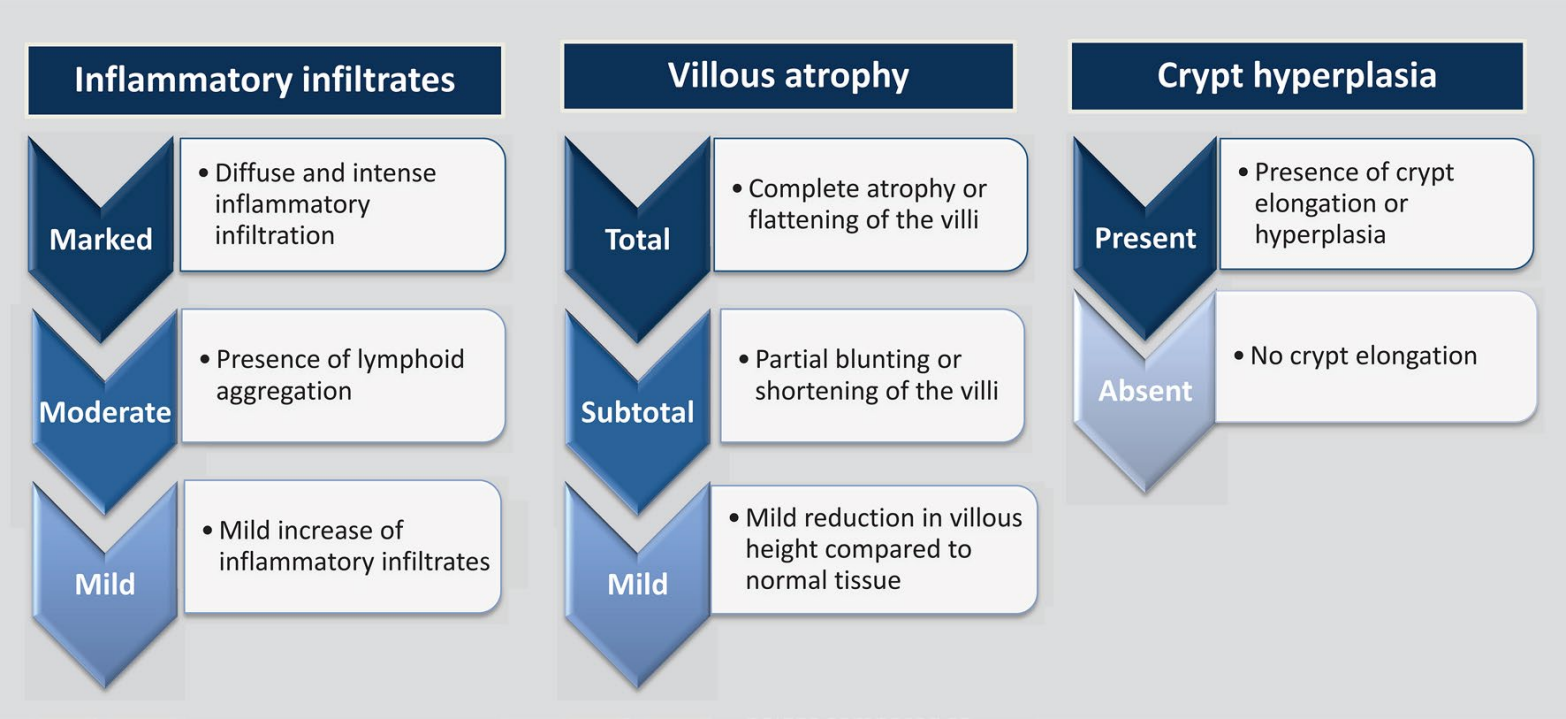
Schematic presentation for assessment of the histological alterations of intestinal mucosa.

As reported in several studies^2,6,9,17,18^, morphometric parameters of intestinal histology included measures of villous and crypt remodeling and infiltration of inflammatory infiltrates in the lamina propria. Villous changes included villous blunting or atrophy, which was defined as flattening of the mucosal surface secondary to the shortening and blunting of the intestinal villi. Total villous atrophy was defined as complete atrophy or flattening of the villi with a villous to crypt (V:C) ratio between 0:1 and 1:1. Subtotal villous atrophy referred to partial blunting or shortening of the villi and mild villous atrophy referred to mild reduction of villous height than normal^19^. Changes in crypt denoted to elongation or hyperplasia of the crypts and was defined as an increase in the length of crypts and a reduction in the normal crypt to villous ratio^19^. For estimating inflammatory infiltrates in the lamina propria, even in the absence of active inflammation, the lamina propria might consist of plasma cells and lymphocytes^19^, and slides containing the presence of an excess of these infiltrates than normal were only taken into consideration. As judged by the eye, presence of inflammatory infiltrates was further categorized into marked, moderate and mild forms. A marked infiltration denoted to an intense and diffuse inflammatory infiltration clearly distinguishable on naked eye under the microscope. Moderate infiltration referred to the presence of a lymphoid aggregation or follicle and mild infiltration referred to a higher number of infiltrates compared to normal. Figure 2 shows the schemes used to assess the morphological alterations of intestinal mucosa.

An increased IEL count in an otherwise normal duodenal biopsy specimen may be associated with certain immunological disorders, use of NSAIDs, lymphocytic/collagenous colitis, bacterial overgrowth and gluten sensitive enteropathy^19^. More than 30 IELs/100 enterocytes was diagnosed as intraepithelial lymphocytosis^19^. IEL count was done by counting the number of lymphocytes per 20 enterocytes present on a random villous tip, and then summing up the numbers of these lymphocytes from such 5 random villi^19^. That is, 20 enterocytes multiplied by 5 villi resulting in the number of lymphocytes per 100 enterocytes. Subjective morphologic analysis of the mucosal surface architecture was carried out in LEICA DM 1000 LED microscope.

### Statistical analyses

Mean values, standard deviation (SD) and 95% confidence intervals (CI) of means were used to describe the distribution and prevalence. Bivariate analysis was performed to quantify the relation between malnutrition and histological features. Demographic characteristics of the study population were described by frequency with proportions for categorical variables, mean with standard deviation for symmetric continuous variables and median with inter-quartile ranges for asymmetric continuous data. Group-wise comparison of the quantitative asymmetric variables in the demographic characteristics was done using the Kruskal–Wallis test. Pearson’s chi-square test or Fisher’s exact test, whichever applicable, was used for qualitative variables and were applied to compare the baseline characteristics between the malnourished and control adults, distribution of duodenitis and comparison of histological features between the malnourished and healthy adults. Statistical significance was defined as a p-value of less than 0.05. Statistical analyses were performed using SPSS version 20.0 (IBM).

## Results

A total of 64 adults (37 malnourished adults, and 27 age and sex matched adult controls) with mean age of 26 ± 6 years underwent UGI endoscopy. The mean age of the malnourished group was 25 ± 6 years, while the mean age of the control group was 27 ± 6 years. Overall, 39 of the 64 adults (60.9%) were female. The percentage of females in the malnourished and the control group was 62% (23/37) and 59% (16/27), respectively. Table 1 shows the demographic characteristics of the study population.

**Table 1:** Demographic characteristics of the malnourished and control adults.

Variables	Adult malnourished († n = 37)	Adult control († n = 27)	Total († n = 64)	† P-value
Age in years, mean (†SD)	25.4 (5.7)	27.5 (5.6)	26.4 (5.7)	0.10
Sex (Female), n (%)	23 (62.2%)	16 (59.3%)	39 (60.9%)	0.81
†BMI in kg/m^2^, mean (SD)	17.4 (1.05)	23.5 (2.94)	21.2 (3.82)	** < 0.001**
†Hb% in gm/dL, mean (SD)	12.6 (1.6)	N/A	N/A	**–**
**Level of education**				
Illiterate, n (%)	4 (10.8%)	2 (7.4%)	06 (09.4%)	0.36
Primary, n (%)	5 (13.5%)	6 (22.3%)	11 (17.2%)	
Secondary, n (%)	24 (64.9%)	11 (40.7%)	35 (54.7%)	
Higher secondary, n (%)	3 (8.1%)	3 (11.1%)	06 (09.4%)	
Graduate, n (%)	1 (2.7%)	5 (18.5%)	06 (09.4%)	
**Occupation**				
Service	4 (10.8%)	4 (14.9%)	8 (12.5%)	0.73
Business	3 (8.1%)	2 (07.4%)	5 (7.8%)	
Labourer	4 (10.8%))	3 (11.1%)	7 (10.9%)	
Driver	3 (8.1%)	0 (00.0%)	3 (4.8%)	
Housewife	13 (35.2%)	11 (40.7%)	24 (37.5%)	
Factory worker	10 (27.0%)	5 (18.5%)	15 (23.4%)	
Others	0 (00.0%)	2 (07.4%)	2 (3.1%)	
Monthly family income († USD)*, mean (SD)	162.3 (106.9)	173.9 (135.7)	167.1 (118.5)	0.71

*1 USD = 85.07 BDT was used as conversion rate.

^†^*n* Number of respondents, *P-value* Significance level, *SD* Standard deviation, *BMI* Body mass index, *Hb%* Hemoglobin, *USD* United States Dollars.

Majority of the adults from both malnourished and control groups had chronic non-specific duodenitis on histopathology. The histological features of malnourished adults significantly pointed towards an active inflammation, which is chronic active duodenitis (p = 0.003). However, the inclination was significantly more towards the non-active form of chronic duodenitis (p = 0.007) in controls. There were 2 cases from the malnourished cohort who had specific duodenitis, one of them having diagnosed as celiac disease and the other with immune-proliferative small intestinal disease (IPSID). A small number of malnourished adults also had raised IEL count. Table 2 shows the distribution of duodenitis based on the operational classification.

**Table 2:** Distribution of duodenitis based on the operational classification.

Duodenitis	Adult cohort		
	Malnourished †n = 37 †n (%)	Control †n = 27 †n (%)	†P-value
Based on known pathology			
Normal	0 (0%)	1 (6%)	0.42
Specific duodenitis	2 (5%)	0 (0%)	0.50
Non-specific duodenitis	35 (95%)	26 (96%)	1.00
Based on tissue morphology			
Normal	0 (0%)	1 (6%)	0.42
Chronic duodenitis	12 (32%)	18 (66%)	**0.007**
Chronic active duodenitis	25 (68%)	8 (30%)	**0.003**
Intraepithelial lymphocytosis (†IEL > 30)	2 (5%)	0 (0%)	0.50

^†^*n* Number of respondents, *P-value* Significance level, *IEL* Intraepithelial lymphocytosis.

*Tests used*; Pearson’s Chi-Square test, Fisher’s Exact Test.

**Figure 3:**
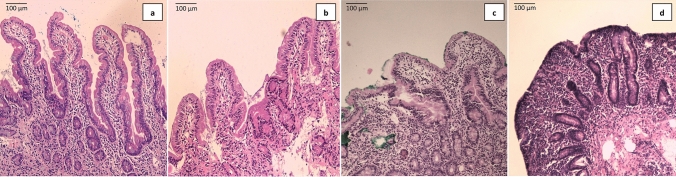
Histological images of villous height obtained using hematoxylin and eosin (H&E) stain. Representative pictures of—(**a**) Normal villous architecture (**b**) Mild villous atrophy (**c**) Subtotal villous atrophy (**d**) Total villous atrophy with crypt hyperplasia.

**Figure 4:**
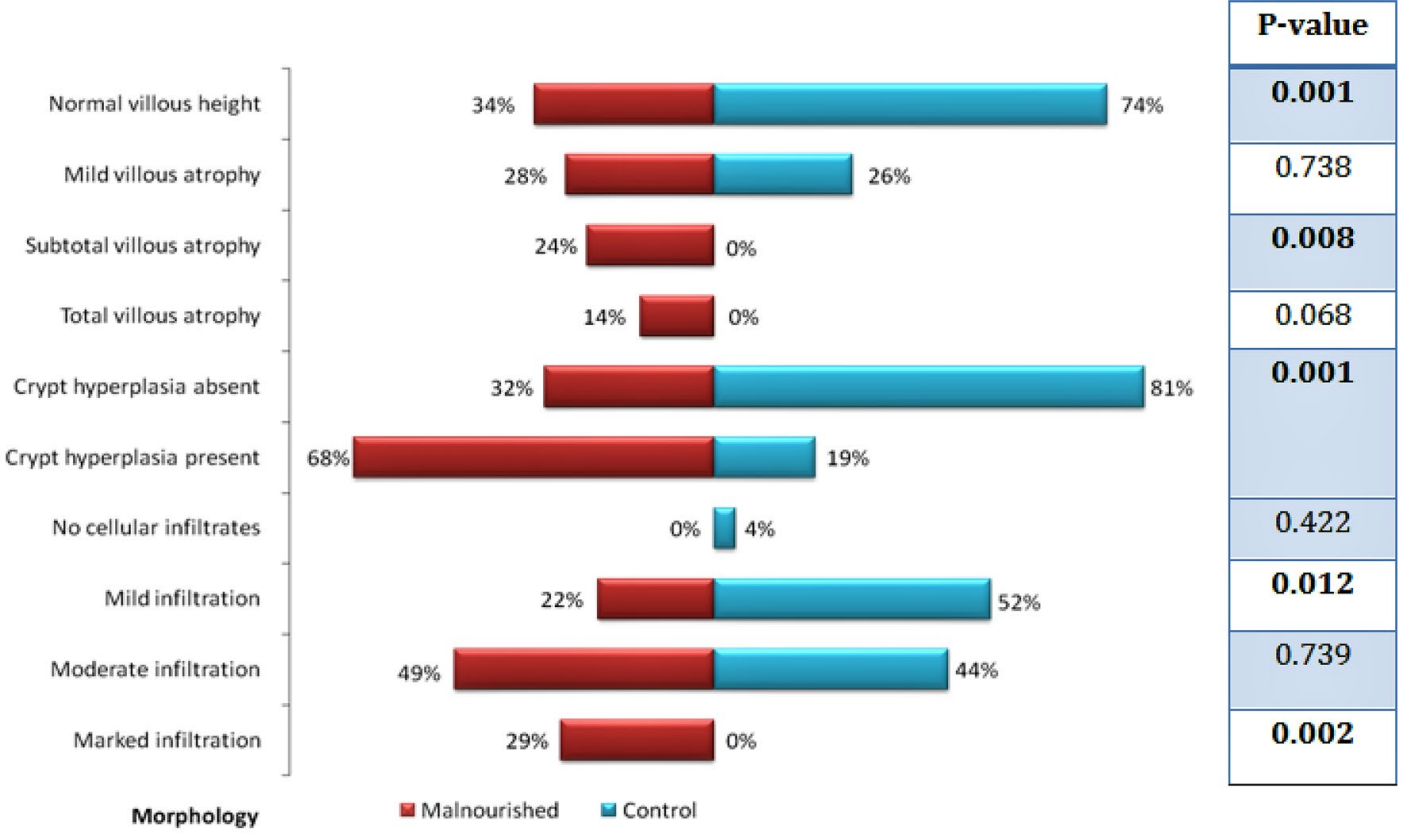
Comparison of histological features between malnourished and healthy adults. *P-value* Significance level. *Tests used*; Pearson’s Chi-Square test, Fisher’s exact test.

Majority of the adults from the control group had normal villous height (20/27) as well as crypt depth (22/27). On the other hand, 24% (9/37) of the malnourished cases had subtotal villous atrophy, 14% (5/37) of the malnourished cases had total villous atrophy and 68% (25/37) of the malnourished cases had crypt hyperplasia. Representative histological images of normal villous architecture, mild villous atrophy, subtotal villous atrophy and total villous atrophy with crypt hyperplasia obtained using hematoxylin and eosin (H&E) stain are shown in Fig. 3a–d respectively. Though mild lymphocytic infiltration was present in more than half (14/27) of the controls, marked inflammatory infiltration was present in 29% (11/37) of the malnourished group compared to none in the control group. A statistically significant difference (p < 0.05) was observed between malnourished and control groups in terms of villous height, crypt depth and inflammatory infiltration (Fig. 4).

## Discussion

Our study revealed a high prevalence of chronic non-specific duodenitis diagnosed by histopathology in Bangladeshi adults, irrespective of nutritional status. The finding is novel as there is no nationally representative data on duodenitis for this age group. A thorough literature search yielded very limited data on the prevalence of duodenitis from this geographic region. Though several studies exist that have investigated pathologies of the stomach and esophagus^20^, very few reported duodenal pathologies^20^. The findings conform to a recent study which also showed high prevalence of duodenitis in two-year-old chronic malnourished slum-dwelling children in Dhaka^21^. A study in Sweden reported 32% prevalence of duodenitis in adults with GI symptoms^22^, however, those participants were well-nourished and underwent UGI endoscopy for diagnostic purposes, whereas more than half of the population in the current study were malnourished and asymptomatic.

The most common etiology for duodenitis in Western countries is celiac disease (CD), accounting for as high as 32%^23^. In our study, only one malnourished adult, who also fell in the category of specific duodenitis, was found positive for CD, and the IEL count, which is a key histopathologic marker of CD^24^, was also insignificant in the studied population. Due to ethical reasons, the malnourished adults who responded to nutritional intervention could not be biopsied. Biopsy samples could not be collected from absolutely healthy, asymptomatic individuals either, due to similar ethical considerations. The control group considered in the present study consisted of age and sex-matched well-nourished (BMI > 18.5) adults suffering from functional dyspepsia diagnosed by Rome III criteria who had no evidence of any organic diseases on endoscopy, clinical and biochemical assessment and were otherwise healthy^12^. A study conducted on Dutch population showed that 83% patients with functional dyspepsia with no evident organic disease had chronic non-specific duodenitis on histopathology^25^, which is similar to our findings in this study.

Comparing the histological characteristics of both malnourished and control groups showed significant difference in villous height, crypt depth and inflammatory infiltration. The malnourished adult group had significantly higher frequency of subtotal villous atrophy, crypt hyperplasia and marked cellular infiltration (p < 0.05) than the controls. On the other hand, the control group had significantly higher prevalence of normal villous height, normal crypt depth and mild form of inflammation compared to the malnourished group (p < 0.05). Most importantly, the frequency of chronic active duodenitis was significantly high in malnourished population (p < 0.05) compared to the control group. As already mentioned, these malnourished adults failed to respond to nutritional therapy and were also negative for any other secondary causes of malnutrition and thus were considered as probable cases of EED. The active feature is found in the setting of a relatively long term chronic inflammatory condition, which in turn is characterized by features like atrophied villi, hyperplastic crypts, inflammatory cellular infiltrates etc. This might in part explain the reason of the high frequency of active duodenitis in the malnourished cohort. Then again, studies relating EED biomarkers with the histopathology results are required to establish this as a fact.

### Limitations

This study was conducted as a part of the Bangladesh Environmental Enteric Dysfunction (BEED) study and the target population of the present study were malnourished (BMI < 18.5 kg/m^2^) and well-nourished (BMI > 18.5) adults aged 18–45 years. As collection of small intestinal biopsy samples from absolutely healthy, well-nourished, asymptomatic individuals without an evident clinical condition is ethically infeasible, the control group considered in the present study consisted of age and sex-matched well-nourished apparently healthy adults suffering from functional dyspepsia with no evidence of any organic diseases on endoscopy and clinical assessment, who underwent upper GI endoscopy for evaluation of dyspepsia. Such participants diagnosed with peptic ulcer disease, malignancy etc. during the endoscopy procedure were excluded. For similar ethical considerations, the malnourished adults who responded to nutritional intervention could not be included either. Measurement of the exact point-to-point height of the villi was not possible. Association of EED markers with gut histology was also not explored. This remained a limitation of the study.

## Conclusion

The prevalence of chronic non-specific duodenitis in Bangladeshi adults, irrespective of nutritional status, was high. Malnourished adults had significantly higher frequency of subtotal villous atrophy, crypt hyperplasia and marked cellular infiltration than the controls, whereas, healthy adults had significantly higher prevalence of normal villous height, normal crypt depth and mild form of inflammation compared to the malnourished group. The prevalence of chronic active duodenitis was significantly high in the malnourished adults.

## Data Availability

The dataset generated and analyzed during the current study is available from the corresponding author on reasonable request.
